# Effects of endurance training on the expression of host proteins involved in SARS‐CoV‐2 cell entry in C57BL/6J mouse

**DOI:** 10.14814/phy2.15014

**Published:** 2021-09-14

**Authors:** Yuki Tamura, Eunbin Jee, Karina Kouzaki, Takaya Kotani, Koichi Nakazato

**Affiliations:** ^1^ Graduate School of Health and Sport Science Nippon Sport Science University Tokyo Japan; ^2^ Research Institute for Sport Science Nippon Sport Science University Tokyo Japan; ^3^ Faculty of Sport Science Nippon Sport Science University Tokyo Japan; ^4^ Graduate School of Medical and Health Science Nippon Sport Science University Tokyo Japan; ^5^ Faculty of Medical Science Nippon Sport Science University Tokyo Japan

**Keywords:** COVID‐19, endurance exercise, endurance training, mouse, SARS‐CoV‐2

## Abstract

The coronavirus disease 2019 (COVID‐19) pandemic, caused by severe acute respiratory syndrome coronavirus 2 (SARS‐CoV‐2), is threatening people's lives and impacting their health. It is still unclear whether people engaged in physical activity are at an increased risk of SARS‐CoV‐2 infection and severe forms of COVID‐19. In order to provide data to help answer this question, we, therefore, investigated the effects of endurance training on the levels of host proteins involved in SARS‐CoV‐2 infection in mice. Eight‐week‐old C57BL/6J mice were subjected to treadmill running (17–25 m/min, 60–90 min, 5 sessions/week, 8 weeks). After the intervention, the levels of angiotensin‐converting enzyme 2 (ACE2; host receptor for SARS‐CoV‐2), transmembrane protease serine 2 (TMPRSS2; host protease priming fusion of SARS‐CoV‐2 to host cell membranes), FURIN (host protease that promotes binding of SARS‐CoV‐2 to host receptors), and Neuropilin‐1 (host coreceptor for SARS‐CoV‐2) were measured in 10 organs that SARS‐CoV‐2 can infect (larynx, trachea, lung, heart, jejunum, ileum, colon, liver, kidney, and testis). Six organs (heart, lung, jejunum, liver, trachea, and ileum) showed changes in the levels of at least one of the proteins. Endurance training increased ACE2 levels in heart (+66.4%), lung (+37.1%), jejunum (+24.7%) and liver (+27.4%), and FURIN in liver (+17.9%) tissue. In contrast, endurance training decreased Neuropilin‐1 levels in liver (−39.7%), trachea (−41.2%), and ileum (−39.7%), and TMPRSS2 in lung (−11.3%). Taken together, endurance training altered the levels of host proteins involved in SARS‐CoV‐2 cell entry in an organ‐dependent manner.

## 1 INTRODUCTION

Coronavirus disease 2019 (COVID‐19), which can result from infection with severe acute respiratory syndrome coronavirus 2 (SARS‐CoV‐2), is a pandemic and a serious global threat to human health and individual survival. The SARS‐CoV‐2 virus contains a single‐stranded RNA genome that encodes structural and nonstructural proteins necessary for the viral life cycle. Among the structural proteins, the spike glycoproteins play a particularly important role in cell entry (Mittal et al., 2020). These spike proteins are cleaved into an S1/S2 site by the host protease FURIN. Consequently, the receptor‐binding domain (RBD) of the S1 subunit binds to the host's angiotensin‐converting enzyme 2 (ACE2), and an area close to the FURIN cleavage site of the S1 subunit binds to Neuropilin‐1 in the host plasma membrane (Cantuti‐Castelvetri et al., 2020; Hoffmann et al., 2020; Wu et al., 2020,). Subsequently, cleavage of the S2 site by the host transmembrane protease serine 2 (TMPRSS2) exposes the membrane fusion peptide of the SARS‐CoV‐2 spike glycoprotein, which leads to the priming of fusion between the virus and host cell membranes and the progression of cell entry by the virus (Hoffmann et al., 2020). Based on these infection mechanisms, ACE2, TMPRSS2, FURIN, and Neuropilin‐1 are considered to be critical host factors for cell entry by SARS‐CoV‐2. Studies of cultured cells have shown that genetic or pharmacological inhibition of the expression or activity of these host factors can attenuate host cell entry by SARS‐CoV‐2 (Bestle et al., 2020; Cantuti‐Castelvetri et al., 2020; Hoffmann et al., 2020; Wu et al., 2020). Therefore, changes in the expression of these host proteins may affect SARS‐CoV‐2 infectivity as well as symptom patterns and the risk of COVID‐19 severity.

Rapid lifestyle changes in response to the COVID‐19 pandemic have reportedly led to a decrease in daily physical activity and an increase in sedentary behavior (Aubertin‐Leheudre & Rolland, 2020). Decreased physical activity contributes to an increased risk of various chronic diseases, such as obesity, diabetes, and cardiovascular disease (Booth et al., 2012), therefore, it is highly beneficial to maintain sufficient physical activity and exercise levels, even during a pandemic. To date, it remains unknown whether exercise habits modulate host susceptibility to SARS‐CoV‐2 infection and the risk of a severe COVID‐19 course. Answering these essential questions will help us to estimate the health risks to athletes and to prescribe safer exercise regimes in the COVID‐19 pandemic. In order to provide data to help answer this question, we, therefore, aimed to elucidate the effects of endurance training on key host factors involved in SARS‐CoV‐2 infection (ACE2, TMPRSS2, FURIN, and Neuropilin‐1) using a mouse model in this study. It has been shown that SARS‐CoV‐2 can infect organs other than the respiratory system, and people with COVID‐19 have had a wide range of symptoms (Mao et al., 2020; Salamanna et al., 2020). Therefore, we analyzed the organs that have been reported to be susceptible to SARS‐CoV‐2 infection: larynx, trachea, lung, heart, liver, jejunum, ileum, colon, testis, and kidney.

## 2 MATERIALS AND METHODS

### 2.1 Ethical approval

All animal experiments were approved by the Animal Experimental Committee of Nippon Sport Science University (no. 020‐A05). The authors have read, and all experiments complied with, the policies and regulations of the Fundamental Guidelines for Proper Conduct of Animal Experiments and Related Activities in Academic Research Institutions published by the Ministry of Education, Culture, Sports, Science and Technology, Japan (no. 71, 2006).

### 2.2 Experimental animals

Twelve 8‐week‐old male C57BL/6J mice (CLEA Japan) were randomly divided into a sedentary control (*n* = 6) and endurance training group (*n* = 6). Based on our previous work (Tamura et al., 2014) and the preliminary validation, the sample size was sufficient to detect adaptations with endurance training, at least in skeletal muscle. The mice were housed (6 mice/cage, cage size 19.7 × 37.5 × 13.3 cm) in a 12‐h light/dark cycle (18:00–06:00 h) at 22℃. They had access to standard chow (CE‐2, CLEA Japan) and water ad libitum.

### 2.3 Endurance training protocol

Among the many endurance training methods, treadmill running was adopted in this study because of the priority to accurately specify and report the experimental conditions (i.e., intensity, time, and frequency). Mice in the endurance training group were subjected to endurance running on a motor‐driven treadmill. The mice performed treadmill running 5 days/week for 8 weeks. During the training period, workloads (speed and/or duration) in each exercise session were progressively increased (weeks 1 and 2: 17 m/min, 60 min; weeks 3 and 4: 20 m/min, 60 min; weeks 5 and 6: 25 m/min, 60 min; and weeks 7 and 8: 25 m/min, 90 min).

### 2.4 Tissue collection

Twenty‐four hours after the final exercise session, the mice were sacrificed with cervical dislocation. Gastrocnemius muscle, larynx, trachea, lung, heart, liver, jejunum, ileum, colon, kidney, testis, and inguinal adipose tissue (iWAT) were collected and washed in PBS. Tissues were frozen in liquid nitrogen and stored at −80℃ until used in further assays.

### 2.5 Protein extraction and western blotting

Protein extraction and western blotting analysis were performed as described previously (Tomiya et al., 2019; Wakabayashi et al., 2020). Briefly, tissues were homogenized in radioimmunoprecipitation assay buffer (188‐02453, Fujifilm Wako Pure Chemical Corporation) containing a protease and phosphate inhibitor cocktail (169‐26063/167‐24381, Fujifilm Wako Pure Chemical Corporation). The protein concentration was measured using the bicinchoninic acid (BCA) method (295‐78401, Fujifilm Wako Pure Chemical Corporation). Equal amounts (2.5–10 μg) of protein were separated using standard SDS‐PAGE on 10% and 12% (w/v) TGX polyacrylamide gels (161‐0173/161‐0175, Bio‐Rad) and transferred to polyvinylidene difluoride membranes (IPVH00010, Merck Millipore). Protein transfer was confirmed by staining with Ponceau S (33427.01, SERVA Electrophoresis GmbH). The membranes were blocked with blocking reagent (NYPBR01, Toyobo Co., Ltd.) for 1 h and incubated for 1 h with primary antibodies diluted in Can Get signal reagent 1 (NKB‐101, Toyobo Co., Ltd.). The antibodies used in this study are listed below. After incubation, the membranes were washed with Tris‐buffered saline containing 0.01% Tween 20 (TBST,T9142, Takara Bio Inc.), then incubated for 1 h at room temperature with the secondary antibodies (7074/7076, Cell Signaling Technology) diluted in Can Get signal reagent 2 (NKB‐101, Toyobo Co., Ltd.) and washed again with TBST. Chemiluminescent reagents (SuperSignal West Pico Chemiluminescent Substrate; Thermo Fisher Scientific) were used for protein detection. The blots were scanned and quantified using ChemiDoc XRS (170‐8071, Bio‐Rad) and Quantity One (170‐9600, version 4.5.2, Windows; Bio‐Rad), and we used the Ponceau S signal (25–150 kDa) intensity as a loading control.

### 2.6 Primary antibodies

The following antibodies were used: Myoglobin (1:3000, 16048‐1‐AP, Proteintech); cytochrome c oxidase subunit IV (COX IV; 1:1000, 4844, Cell Signaling Technology); glucose transporter 4 (GLUT4; 1:1000, 66846‐1‐IG, Proteintech); monocarboxylate transporter 1 (MCT1; 1:1000, 20139‐1‐AP, Proteintech); MCT4 (1:3000, 27787‐1‐AP, Proteintech); uncoupling protein 1 (UCP1; 1:1000, ab23841, Abcam); ACE2 (1:2000, 21115‐1‐AP, Proteintech); TMPRSS2 (1:2000, NBP3‐00492, Novus Biologicals); FURIN (1:2000, 18413‐1‐AP, Proteintech); and Neuropilin‐1 (1:2000, 3725, Cell Signaling Technology).

### 2.7 Statistical analysis

Data were expressed as mean ± SE for individual plots. Differences between the control and endurance training group were examined using the Mann–Whitney U test, and statistical significance was defined as *p* < 0.05. Statistical tests were performed using GraphPad Prism (Version 9.0.0, Mac, GraphPad Software).

## 3 RESULTS

### 3.1 Validation and profile of endurance training

**FIGURE 1:**
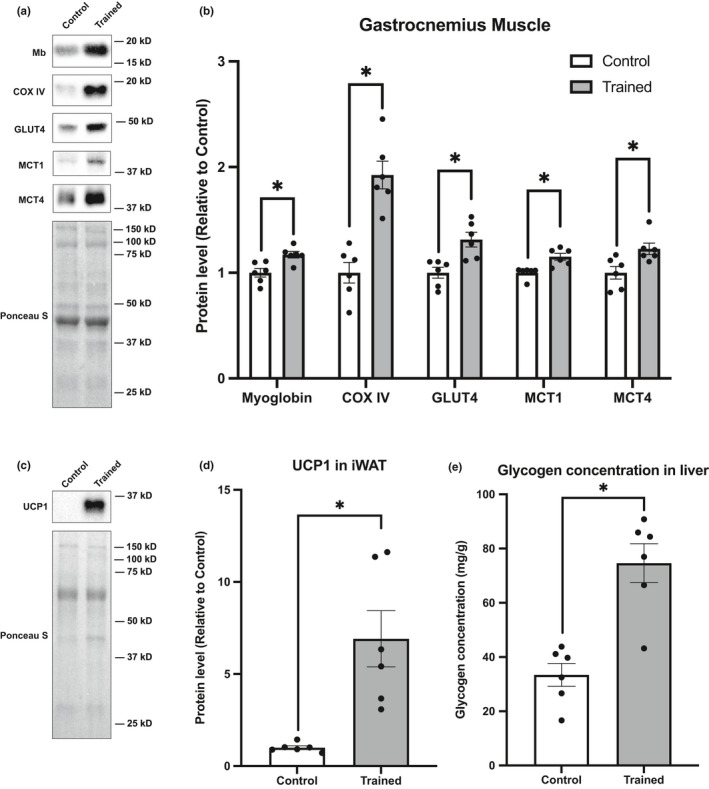
Validation and profiles of endurance training model. (a) Representative blots and (b) protein content of myoglobin, COX IV, GLUT4, MCT1, and MCT4 in gastrocnemius muscle. (c) Representative blots and (d) protein content of UCP1 in iWAT. (e) Glycogen concentration in liver. Data are expressed as mean ± SE. Differences between control and endurance training group were examined using the Mann–Whitney U test. Statistical significance was defined as *p* < 0.05

First, we evaluated whether adaptation by endurance training was sufficiently induced and the profile of adaptations. A slight decrease in body weight and a decrease in white adipose tissue weight were observed (Table 1). On the other hand, skeletal muscle weight was not affected by the endurance training in this study (Table 1). Eight weeks of endurance training induced significant oxidative adaptations in the skeletal muscle (Figure 1a,b), such as increased levels of myoglobin (+17.1%), biomarkers of mitochondrial content (COX IV [+92.4%]), and substrate transporters (GLUT4 [+31.4%], MCT1 [+15.1%], and MCT4 [+21.0%]). In addition to skeletal muscle adaptations, we also observed systemic adaptations to the endurance training; specifically, there was a clear induction of browning of iWAT (i.e., UCP1 induction [Figure 1c,d; +591.4%]). Moreover, we found that endurance training increased the glycogen concentration of the liver (Figure 1e; +123.4%). These data clearly support that skeletal muscle and non‐skeletal organ adaptations are sufficiently induced by endurance training in this work. These findings strongly warrant a subsequent analysis of protein‐based adaptations involved in SARS‐CoV‐2 cell entry in various tissues.

**TABLE 1:** Physical characteristics after endurance training intervention

	Control	Trained	*p* value
Body weight (g)	29.32 ± 0.63	27.25 ± 0.53	0.041
Soleus muscle weight (mg)	10.25 ± 0.37	8.93 ± 0.14	0.065
Gastrocnemius muscle weight (mg)	137.4 ± 2.25	132.3 ± 2.60	0.241
Inguinal adipose tissue weight (mg)	245.8 ± 12.5	201.7 ± 8.82	0.026
Epididymal adipose tissue weight (mg)	442.4 ± 15.7	243.1 ± 11.2	0.002

### 3.2 Effects of endurance training on the levels of proteins involved in SARS‐CoV‐2 cell entry in select cardiorespiratory organs

**FIGURE 2:**
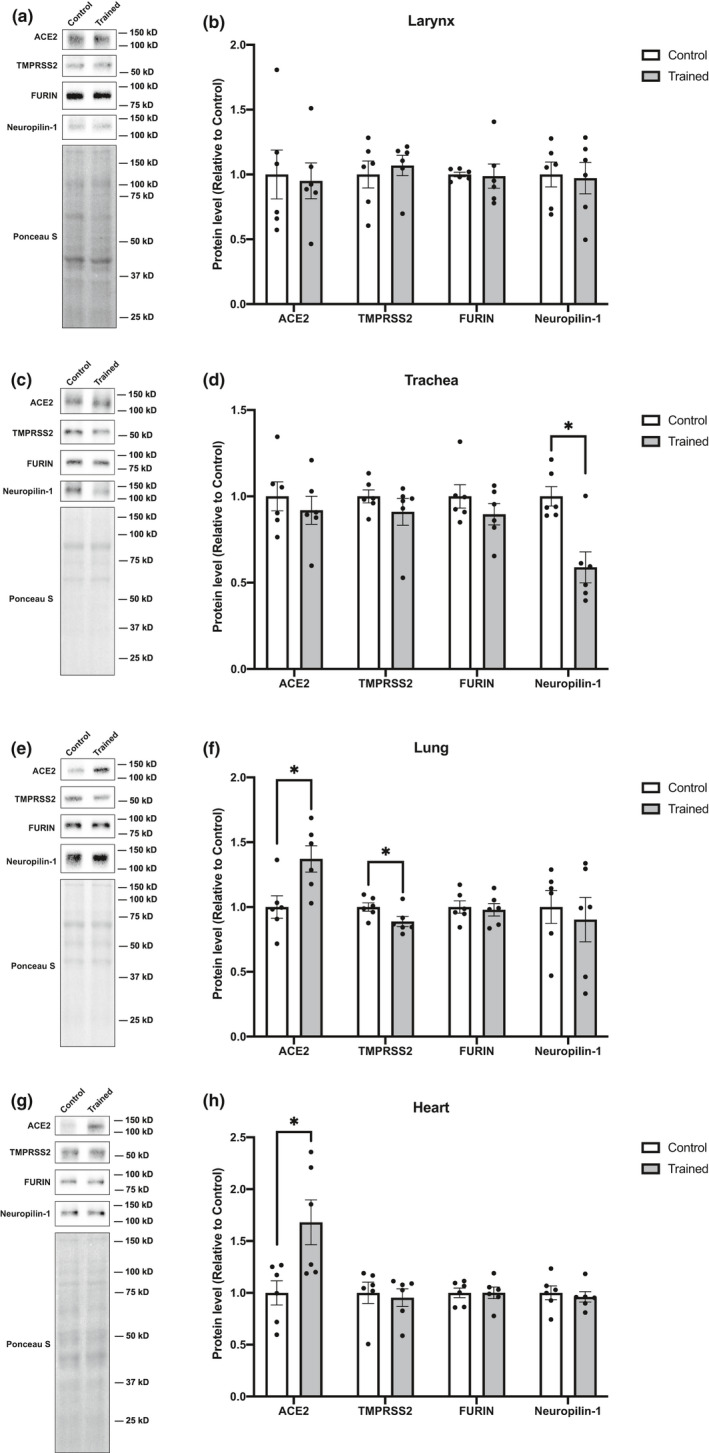
Effects of endurance training on ACE2, TMPRASS2, FURIN, and Neuropilin‐1 protein levels in cardiorespiratory organs. Representative blots and protein content of ACE2, TMPRSS2, FURIN, and Neuropilin‐1 in (a, b) larynx, (c, d) trachea, (e, f) lung, and (g, h) heart. Data are expressed as mean ± SE. Differences between control and endurance training group were examined using the Mann–Whitney U test. Statistical significance was defined as *p* < 0.05

We analyzed protein levels in the cardiorespiratory organs such as: larynx, trachea, lung, and heart. With endurance training, there was no detectable change in protein levels measured in the larynx (Figure 2a,b). However, endurance training decreased Neuropilin‐1 levels in the trachea (Figure 2c,d; −41.2%); increased the content of ACE2 in the lungs (Figure 2e,f; +37.1%); and slightly, but statistically significantly, reduced TMPRSS2 levels (Figure 2e,f; −11.3%). ACE2 protein levels in the heart were increased by endurance training (Figure 2g,h; +66.4%), but the levels of TMPRSS2, FURIN, and Neuropilin‐1 remained unaffected (Figure 2g,h).

### 3.3 Effects of endurance training on the levels of proteins involved in SARS‐CoV‐2 cell infection in select digestive organs

**FIGURE 3:**
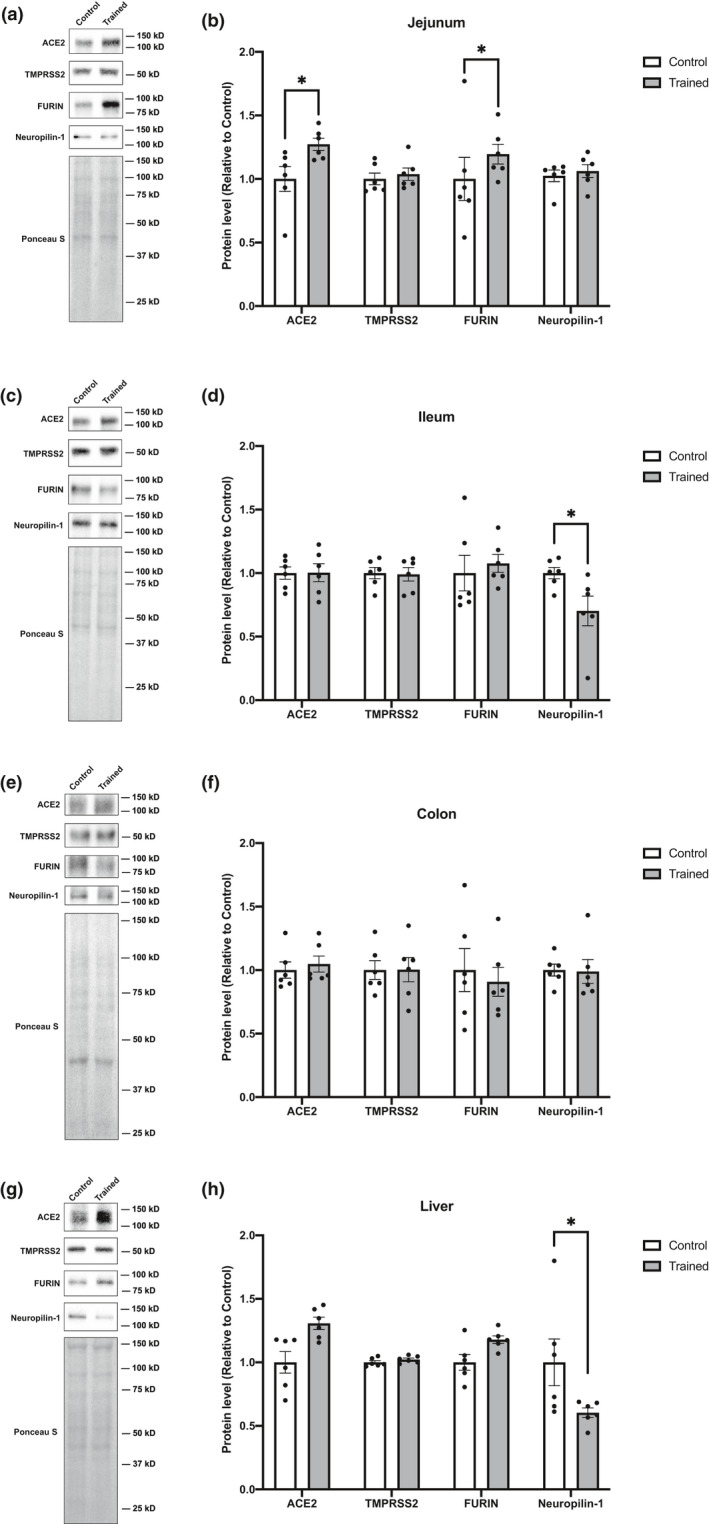
Effects of endurance training on ACE2, TMPRASS2, FURIN, and Neuropilin‐1 protein levels in digestive organs. Representative blots and protein content of ACE2, TMPRSS2, FURIN, and Neuropilin‐1 in (a, b) jejunum, (c, d) ileum, (e, f) colon, and (g, h) liver. Data are expressed as mean ± SE. Differences between control and endurance training group were examined using the Mann–Whitney U test. Statistical significance was defined as *p* < 0.05

We next analyzed the levels of proteins in the digestive organs such as: jejunum, ileum, colon, and liver. In the jejunum, ACE2 protein content was increased with endurance training (Figure 3a,b; +24.7%). Whereas no statistically significant changes in TMPRSS2, FURIN, and Neuropilin‐1 levels were detected (Figure 3a,b). Mice subjected to endurance training showed decreased levels of Neuropilin‐1 in the ileum (Figure 3c,d; −30.6%), but no changes in ACE2, TMPRSS2, or FURIN (Figure 3c,d). Endurance training had no effect on the levels of ACE2, TMPRSS2, FURIN, or Neuropilin‐1 in the colon (Figure 3e,f). In the liver, ACE2 and FURIN protein levels were significantly increased (Figure 3g,h; ACE2 [+27.4%]; FURIN [+17.9%]), and Neuropilin‐1 levels were decreased (Figure 3g,h, −39.7%).

### 3.4 Effects of endurance training on the levels of proteins involved in SARS‐CoV‐2 cell infection in the urogenital system

**FIGURE 4:**
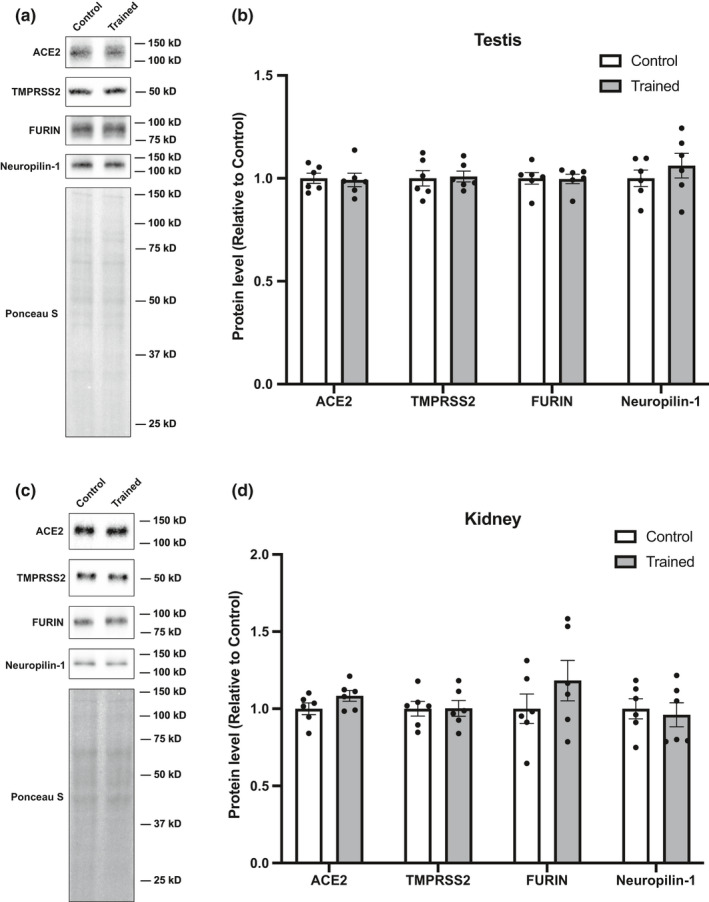
Effects of endurance training on ACE2, TMPRASS2, FURIN, and Neuropilin‐1 protein levels in urogenital organs. Representative blots and protein content of ACE2, TMPRSS2, FURIN, and Neuropilin‐1 in (a, b) testis, and (c, d) kidney. Data are expressed as mean ± SE. Differences between control and endurance training group were examined using the Mann–Whitney *U* test. Statistical significance was defined as *p* < 0.05

In addition to examining the respiratory and digestive organs, we assessed changes in host factor proteins with endurance training in urogenital organs (testis and kidney). When we examined the testis (Figure 4a,b) and kidneys (Figure 4c,d), we found that endurance training did not result in any quantitative changes in any of the proteins analyzed in this study.

## 4 DISCUSSION

Changes in ACE2 expression resulting from endurance training have been studied in several organs in the past (Barretti et al., 2012; Kar et al., 2010; Somineni et al., 2014). Those studies, however, only examined changes in the heart, brain, or kidney from the perspective of blood pressure regulation by the renin–angiotensin system. In contrast, this study aimed to understand the infection processes of SARS‐CoV‐2, thus we examined changes in ACE2 levels in more organs than previously reported. Furthermore, we also examined changes in the concentrations of proteases (TMPRSS2 and FURIN) and a co‐receptor (Neuropilin‐1) required for SARS‐CoV‐2 cell entry. To the best of our knowledge, no study has reported changes in the expression of these proteins in the context of exercise and training. Recently, the possibility that exercise and training may affect susceptibility to SARS‐CoV‐2 infection has been discussed in commentary and review articles (South et al., 2020; Zbinden‐Foncea et al., 2020). However, these descriptions were based on the limited knowledge of exercise training‐induced ACE2 adaptation. Therefore, this study made an important contribution, in that it comprehensively and experimentally revealed the alterations in host factors involved in SARS‐CoV‐2 infection by endurance training.

### 4.1 Physiological and clinical significance of endurance training‐induced changes in host factors involved in cell entry by SARS‐CoV‐2

We evaluated the expression levels of ACE2, TMPRSS2, FURIN, and Neuropilin‐1 in 10 organs in this study, of which six (heart, lung, jejunum, liver, trachea, and ileum) showed changes in the expression levels of at least one of the investigated proteins. It was hypothesized that the efficiency of SARS‐CoV‐2 cell entry in these organs may be modulated by physical activity. The heart, lungs, jejunum, and liver showed increased ACE2 levels following endurance training. Elderly people, smokers, and patients with cardiovascular disease or obesity are at a greater risk for severe COVID‐19 (Chakravarty et al., 2020; Hamer et al., 2020; Nishiga et al., 2020), and these patients, and relevant model animals, have been found to have increased expression of respiratory and cardiovascular ACE2 (Cai et al., 2020; Somineni et al., 2014). Therefore, increased ACE2 is assumed to be a potential explanatory factor for variations in COVID‐19 severity. Our results showed a marked increase in ACE2 levels, especially in the heart, with endurance training. Recently, researchers reported that cardiac muscle inflammation occurs as a sequela in competitive athletes who have been suffering from COVID‐19 (Rajpal et al., 2020). However, a limitation of that study was that a non‐athlete control group was not included, and it was not possible to conclude whether myocarditis was an athlete‐specific or a more common sequela of COVID‐19. However, if myocarditis is a characteristic sequela in athletes, it may be partially explained by an increase in ACE2. Further clinical and basic research will allow us to reach a conclusion on this issue.

Studies of cultured cells have shown that, even in cells with forced/overexpressed ACE2, cell entry by SARS‐CoV‐2 can be partially inhibited by blocking Neuropilin‐1 activity with a neutralizing antibody (Cantuti‐Castelvetri et al., 2020). In contrast to ACE2 adaptation, endurance training decreased the levels of Neuropilin‐1 in the liver, trachea, and ileum. Therefore, if in vitro findings can be interpreted for in vivo conditions, endurance training may attenuate cellular infection by SARS‐CoV‐2 in these organs.

### 4.2 Candidate molecular mechanisms of endurance training‐induced adaptations

While the mechanisms behind the quantitative gene/protein regulation of SARS‐CoV‐2 cell entry are not well understood, the area is being actively studied. Recently, androgen receptor (AR) was reported to be involved in the transcriptional regulation of both ACE2 and TMPRSS2 genes (Qiao et al., 2021). Whether or not AR is involved as a common regulator in the quantitative changes in ACE2 and TMPRSS2 induced by endurance training is currently unknown. However, it is clear from the present study that the changes in ACE2 and TMPRSS2 levels resulting from endurance training are quite different. Therefore, we suggest two possibilities for the adaptation of ACE2 and TMPRSS2 by endurance training: (Aubertin‐Leheudre & Rolland, 2020) AR is not involved or (Barretti et al., 2012) AR is involved, but there are other, more influential, regulators. The regulatory mechanisms of FURIN gene expression, as well as those of ACE2 and TMPRSS2, are not fully understood. A study of myocardial development showed FURIN expression is negatively regulated by NKX2‐5 (Dupays et al., 2019). The regulatory mechanisms of Neuropilin‐1 expression have been studied relatively frequently, and the gene is regulated at the transcriptional level by transcription factors such as neuron restrictive silencer factor, and bromodomain‐containing 4 (Jiang et al., 2007; Kurschat et al., 2006). The effects of endurance training on these candidate regulators have not been previously investigated, and the exercise responsiveness/adaptability of the regulators is completely unknown. Therefore, it is essential to first investigate the involvement of the above‐mentioned transcriptional regulators when exploring and defining the mechanisms in the future.

### 4.3 Limitation of this work and future perspectives on our understanding of the potential alternation of SARS‐CoV‐2 cell infectivity with endurance training

Throughout our study, we found that endurance training altered the expression of host proteins involved in SARS‐CoV‐2 infection, leading to a novel hypothesis that endurance training can change host susceptibility to SARS‐CoV‐2 infection, the pattern of symptom presentation, and the risk of severe disease. To further investigate this hypothesis, it is necessary to conduct experiments in which SARS‐CoV‐2 is transmitted to mice undergoing endurance training. This will allow us to develop more comprehensive conclusions on a broad range of factors, such as the adaptation of host factors involved in SARS‐CoV‐2 cell entry and immune system remodeling with exercise. However, to perform such experiments, several issues need to be resolved. One critical issue hampering research into SARS‐CoV‐2 and COVID‐19 in mice is that SARS‐CoV‐2 does not infect these mammals (Dinnon et al., 2020). This is due to the difference in the amino acid sequence of ACE2 between mouse and human. Mice have been useful as experimental animals in research on COVID‐19, and experimental models involving mice have been actively developed. As a solution, scientists have proposed the use of CRISPR/Cas9 genome‐editing technology to substitute the mouse ACE2 gene with the human ACE2 gene (Gurumurthy et al., 2020). Alternatively, it may be conceivable to use the recently identified mouse‐adapted mutant SARS‐CoV‐2, which can infect mice (Dinnon et al., 2020). Using mouse‐adapted SARS‐CoV‐2, research on the pathogenesis of COVID‐19 and vaccines are being developed (Gu et al., 2020). The development of experimental systems using such techniques will help to answer the questions raised by the findings of this study.

The present study is limited to testing in a single endurance training protocol in young male mice. Differences in the characteristics of the subjects under study (e.g., age, sex, and presence of disease) may alter the baseline protein expression levels and may modify adaptability. As for exercise, whether differences in the target exercise style and parameters (e.g., intensity, duration, and frequency) lead to different adaptations needs to be tested in the future.

## 5 SUMMARY

We aimed to elucidate the effects of endurance training on key host factors involved in SARS‐CoV‐2 infection (ACE2, TMPRSS2, FURIN, and Neuropilin‐1) in mice. Our data demonstrated that endurance training altered the levels of host proteins involved in cell entry by SARS‐CoV‐2 in an organ‐dependent manner.

## CONFLICT OF INTEREST

None to declare.
